# Conversion of Branched-Chain Amino Acids to Corresponding Isoacids - An *in vitro* Tool for Estimating Ruminal Protein Degradability

**DOI:** 10.3389/fvets.2019.00311

**Published:** 2019-09-18

**Authors:** Juha Apajalahti, Kirsi Vienola, Kari Raatikainen, Vaughn Holder, Colm A. Moran

**Affiliations:** ^1^Alimetrics Research Ltd., Espoo, Finland; ^2^Alltech Inc., Nicholasville, KY, United States; ^3^Alltech SARL, Vire, France

**Keywords:** bacterial protein degradation, branched-chain amino acids, branched-chain volatile fatty acids, *in vitro* rumen model, protein supplements

## Abstract

In this paper we describe a study that evaluates the applicability of an *in vitro* fermentation model to assess the resistance of protein supplements to rumen degradation. The protein sources used were: soybean meal (SBM); whey protein (WHEY), which was expected to be rapidly degraded, and yeast-derived microbial protein (YMP), which was proposed to be resistant to rumen degradation. The basal diet was composed of grass silage and a commercial compound feed. The protein supplements were added at three isonitrogenous doses. Fermentation was monitored for 24 h and gas production, volatile fatty acids, lactic acid, and ammonia were analyzed at three timepoints. Protein degradation was estimated by determining the extent to which branched-chain amino acids (BCAA) introduced with the protein supplement were converted to corresponding branched-chain volatile fatty acids (BCVFA). At the highest dose of WHEY, 60% of introduced valine, leucine, and isoleucine was recovered as isobutyric, 2-methylbutyric, and isovaleric acid (products of BCAA decarboxylation and deamination), respectively. The BCVFA detected represented 50% of added BCAA with SBM, but <15% with YMP. Further indications that YMP protein is resistant to degradation were provided by analysis of ammonia. With YMP, the residual ammonia concentration only marginally exceeded that of the cultures with no protein supplementation, while it increased dose-dependently when the vessels were supplemented with WHEY or SBM. This suggests that with WHEY and SBM, the rate of deamination exceeded the rate of ammonia assimilation by bacteria. Residual ammonia and BCVFA, the two indicators of protein fermentation, were strongly correlated. Overall bacterial activity was monitored as yield of gas, volatile fatty acids, and bacteria. These three correlating parameters showed that WHEY only modestly stimulated fermentation, whereas SBM and YMP stimulated fermentation extensively, possibly owing to their higher carbohydrate content. The results presented suggest that the *in vitro* fermentation method was suitable for detecting differences in resistance of protein supplements to rumen degradation and following a full method validation could be a useful tool for diet formulation. The data obtained suggested that YMP was the most resistant and WHEY the most susceptible to degradation.

## Introduction

In monogastric animals dietary protein is hydrolyzed in the stomach and duodenum, and the amino acids and small peptides produced are taken up and used for protein synthesis by the host. In ruminants, dietary protein is mainly utilized by rumen microbes, which themselves serve as the main protein source for the animal. This expands the nutritional diversity and makes it possible for the host to benefit from protein sources that would be poorly digested by the endogenous enzymes of the ruminant. In the abomasum, the relative proportion of protein originating from the feed and microbial protein synthesized in the rumen vary, depending on variables such as physiological status of the animal, diet composition, and daily feed intake (1, 2). Reliance on undegraded feed protein increases when the target yield of the animal is high in terms of milk production or body weight gain (2, 3). In such cases, the overall protein demand of the animal exceeds the protein-producing potential of the rumen microbiota, so additional rumen undegradable protein (RUP) is needed (4). The rumen degradability of dietary protein is dependent on factors such as inherent resistance of proteins to rumen degradation, passage rate of the protein, processing temperature, and intentional protection of proteins by chemical means (1).

The residual concentration of ammonia (NH_3_) in the rumen is determined by the relative rate of NH_3_ production, NH_3_ utilization by bacteria, and NH_3_ uptake across the rumen wall. When amino acids are fermented by bacteria, NH_3_ is a major metabolite but many other metabolites are produced concomitantly. When the branched-chain amino acids (BCAA) valine (Val), leucine (Leu), and isoleucine (Ile) are oxidatively deaminated, they are converted to the branched-chain volatile fatty acids (BCVFA) isobutyric, isovaleric and 2-methylbutyric acid, respectively (5).

Over the years many advanced models have been used to examine microbial degradation of protein in the rumen. Here we describe a novel approach in which BCVFA were used as a quantitative and NH_3_ as a supporting marker for protein degradation. With this method the BCAA content of the fermented protein has to be known since it must be taken into account when the yield of BCVFA is used to estimate the degree of protein degradation. The markers used for overall bacterial fermentation activity were gas production, volatile fatty acids (VFA), lactic acid, and bacterial density.

The objective of the present work was to evaluate a novel concept designed to assess protein degradability. In an *in vitro* test we compared three protein supplements with different expected degrees of resistance to rumen degradation: soybean meal (SBM), whey protein (WHEY) and yeast-derived microbial protein (YMP). Soybean meal is a commonly used protein supplement and was therefore a good benchmark product for the study. WHEY was included as a supplement considered to be readily fermented by bacteria. Many different yeast-derived products are now used as supplements for ruminants. Some bulk yeast biomass products have been used for decades, while new live yeast products, cell walls, and hydrolysates are continually being developed. Yeast-derived microbial protein included in this study is being marketed as a protein supplement and it was expected to be challenging for the rumen bacteria to degrade.

## Materials and Methods

### Diets

The basal diet used as substrate in the fermentation studies was composed of fresh grass silage (energy 10.8 MJ/kg, crude protein 16%) and commercial compound feed for dairy cows (Lypsykrossi, Suomen Rehu Ltd., Finland; energy 12.3 MJ/kg, crude protein 19%), dosed 1:1 on a dry matter basis. The protein supplements tested were added to 800 mg of basal diet, at three doses as specified in Table 1. The doses applied for YMP (DEMP®, Alltech Inc., Nicholasville, KY) were 2, 10, and 30% of the final diet dry matter. The doses of the other protein supplements were isonitrogenous with the doses of YMP. Due to the crude protein content of the three supplements being different, the total amount of dry matter introduced into the fermentation vessels varied accordingly (crude protein content of SBM, WHEY, and YMP was 54.2, 89.2, and 45.8%, respectively).

**Table 1:** Experimental substrates introduced to the vessels used for *in vitro* rumen fermentation^a^.

**Diet**	**Basal substrate**		**Protein supplement dosing**			**BCAA introduced with supplements**		
	**Grass silage (mg DM)^b^**	**Compound feed^c^ (mg DM)^b^**	**mg DM^b^**	**% of diet DM**	**% of diet CP**	**Val (mmol)^b^**	**Ile (mmol)^b^**	**Leu (mmol)^b^**
Control	400	400	–	0.0	0.0	–		–
SBM Dose 1	400	400	13.5	1.7	5.0	0.003	0.002	0.004
SBM Dose 2	400	400	75.3	8.6	23	0.016	0.013	0.023
SBM Dose 3	400	400	290	27	53	0.062	0.052	0.088
WHEY Dose 1	400	400	8.22	1.0	5.0	0.004	0.004	0.006
WHEY Dose 2	400	400	45.7	5.4	23	0.023	0.022	0.036
WHEY Dose 3	400	400	176	18	53	0.088	0.085	0.139
YMP Dose 1	400	400	16.0	2.0	5.0	0.003	0.002	0.003
YMP Dose 2	400	400	89.0	10	23	0.017	0.012	0.019
YMP Dose 3	400	400	343	30	53	0.065	0.047	0.074

a DM, dry matter; CP, crude protein; SBM, soybean meal; WHEY, whey protein; YMP, yeast-derived microbial protein; BCAA, branched-chain amino acids.

b Amount introduced into 40-mL cultures.

c *Commercial compound feed Lypsykrossi for dairy cows (Suomen Rehu Ltd., Finland)*.

Fresh grass silage was chopped into 3–8 mm pieces with a sharp stainless-steel cutting blade. A subsample of chopped silage was taken for dry matter determination, but for the actual fermentation studies fresh silage was used since drying is likely to affect fermentation kinetics. Pellets of the compound feed were crushed, but not ground. Each feed component, including protein supplements, was weighed separately into individual fermentation vessels, to ensure that their ratio in each vessel was exactly the same.

### *In vitro* Fermentation Protocol

Feed components were weighed into the 120-mL serum bottles used as fermentation vessels (exact weights in Table 1). The vessels were then flushed with CO_2_ that had been passed through a hot copper catalyst for O_2_ scavenging, and sealed with thick butyl rubber stoppers. A 36-mL portion of anaerobic and temperature-adjusted (+38°C) buffer solution including phosphate and bicarbonate as the buffering agents (6) was introduced into each fermentation vessel under protective O_2_-free CO_2_ flow. The fistulated cow used as a source of inoculum was lactating and being fed 8 kg dry matter per day of a commercial compound feed (Opti-Maituri 26, Lantmännen Feed Oy, Turku, Finland; energy 12.8 MJ/kg, crude protein 26%) and ~40 kg of grass silage (~13 kg dry matter with energy 10.8 MJ/kg and crude protein 16%). The compound feed used had multiple protein components but no SBM, WHEY, or YMP. Fresh rumen fluid was strained through a 3 mm steel mesh and 4 mL was added in each vessel (10% inoculum). Finally, the vessels were sealed with butyl rubber stoppers and aluminum crimps, and incubated at +38°C in a gyratory shaker at 100 rpm. The exact time of inoculation of each vessel was recorded and taken into account when sampling and stopping the fermentation. Inoculation of the fermentation vessels took place in random order, to avoid any bias resulting from time of inoculation or freshness of the inoculum.

Each one of the 10 dietary treatments were applied in 18 replicate vessels. Gas production was measured by puncturing the rubber stopper with a needle connected to an accurate glass syringe with a sensitive ground plunger, and recording the volume of gas released from the vessels. After 4, 10, and 24 h from inoculation, six replicate vessels of each diet were withdrawn from the study for various analyses. Thus, none of the test vessels was sampled in the middle of fermentation to avoid potential effects on fermentation kinetics.

### Analyses

#### Branched-Chain Amino Acids

Branched chain amino acids in each protein supplement were analyzed by Eurofins Scientific Finland Ltd., using an accredited method described in the Official Journal of the European Union [Commission Regulation EU 152/2009, Section F, Determination of Amino Acids (except tryptophane)]. In the method, amino acids were analyzed by ion exchange chromatography after acid hydrolysis. Branched chain amino acids were of particular interest since their conversion to the corresponding BCVFA was monitored.

#### Short-Chain Fatty Acids

Volatile fatty acids and lactic acid, referred to in combination as short-chain fatty acids (SCFA), were analyzed in six replicate fermentation vessels per treatment at the 4-, 10-, and 24-h time-points. The SCFA were analyzed as free acids, using pivalic acid (Sigma-Aldrich, St. Louis, MO, USA) as an internal standard. For this, 400 μL of fermentation fluid and 2.4 mL of 1.0 mM pivalic acid solution were mixed, vigorously shaken for 5 min, and then centrifuged at 3,000 × *g* for 10 min. Then 800 μL of the supernatant and 400 μL of saturated oxalic acid solution were mixed, incubated at 4°C for 60 min, and centrifuged at 18,000 × *g* for 10 min. The supernatant was analyzed by gas chromatography (Agilent Technologies, Santa Clara, CA, USA) using a glass column packed with 80/120 Carbopack B-DA/4% Carbowax stationary phase, helium as a carrier gas, and a flame ionization detector. The acids quantified were acetic, propionic, butyric, valeric, isobutyric, 2-methylbutyric, isovaleric, and lactic acid.

#### Ammonia

Ammonia in fermentation fluid was analyzed at 4, 10, and 24 h after inoculation. The analysis was performed using a colorimetric method based on the reaction of phenol and hypochlorite with NH_3_, leading to color formation, the intensity of which was measured with a spectrophotometer. The method of Weatherburn (7) modified from the Berthelot reaction (8) was applied.

#### Total Bacteria

Bacteria were analyzed by quantitative real-time PCR at the 4-, 10-, and 24-h time-points. First, bacteria from the fermentation samples were lyzed by a method involving enzymatic, chemical, and mechanical disruption (bead beating) of bacterial cell walls as described previously in detail for ileal digesta samples (9). DNA was purified from the homogenates using phenol-chloroform-isoamyl alcohol extraction the steps of which are described elsewhere in detail (9).

DNA was analyzed by quantitative real-time PCR using an ABI Prism Sequence Detection System 7500 instrument (Life Technologies, Carlsbad, CA, USA). The 16S rRNA gene -targeted primers used for the enumeration of total eubacteria were:

- forward 5′-TCCTACGGGAGGCAGCAGT-3′
- reverse 5′-GGACTACCAGGGTATCTAATCCTGTT-3′

The amplifications were performed with SYBR Green chemistry (Life Technologies, USA) and PCR conditions described previously (10). PCR efficiency was 90–95%. For data analysis, 16S gene copies/mL were log_10_-transformed.

### Statistical Analysis

Data were analyzed using the one-way ANOVA in SPSS (IBM, version 22) with protein source (SBM, WHEY, YMP) and protein dose (Low, Medium, High) as main effects. The effect of protein source and dose on the production of various analytes was established separately for each time point (4, 8, and 24 h). ANOVA *P*-values lower than 0.05 were considered significant. Tukey's *post hoc* test was then used to compare differences between the mean values. Pearson correlation coefficients between the fermentation parameters were calculated using the SPSS software.

## Results

### Effect of Protein Supplementation on Recovery of Protein Fermentation Products

#### Residual Concentration of Branched-Chain Fatty Acids

**Figure 1:**
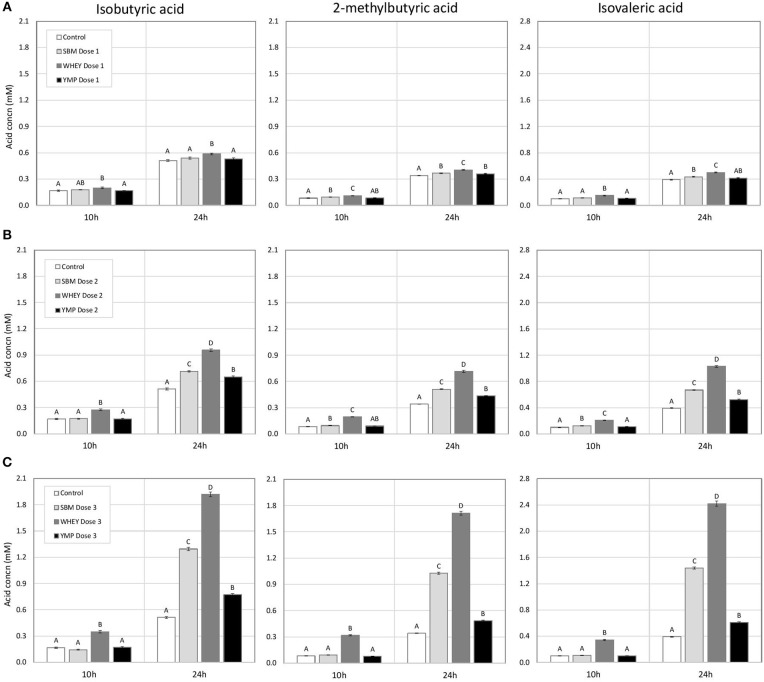
Effect of protein supplements on the concentration of branched chain volatile fatty acids (BCVFA) in *in vitro* rumen fermentation. **(A–C)** Show the concentration of indicated BCVFA in treatments with low, medium, and high dose of isoproteinous supplement, respectively. Error bars indicate standard error of the mean. Different superscripts above bars at each fermentation time indicate significant differences between protein supplements (*P* < 0.05; Tukey HSD test).

The concentration of BCVFA was analyzed at three time-points during the 24-h fermentation. After the first 4 h of fermentation, the treatments had little effect on the concentration of total BCVFA (Table 2). At 10 h, the vessels with WHEY supplement had dose-dependent increases in the concentration of isobutyric, 2-methylbutyric, and isovaleric acid (Figure 1; Table 2). At 24 h, the concentration of BCVFA had increased and the cultures with protein supplements had higher concentrations of BCVFA than the unsupplemented controls. WHEY-supplemented cultures produced more isobutyric, 2-methylbutyric, and isovaleric acid than the other protein supplements at isonitrogenous doses. At the lowest dose of SBM and YMP, no difference in BCVFA concentration was detected between the two. However, in the cultures with medium and high doses, SBM yielded higher residual concentrations of BCVFA than YMP (Figure 1; Table 2).

**Table 2:** Effect of different protein supplements on concentration of various short-chain fatty acids during *in vitro* rumen fermentation^1^.

**Acid (mM)**	**Control**	**Low dose**			**Medium dose**			**High dose**			**SE**	***P*-value**
		**SBM**	**WHEY**	**YMP**	**SBM**	**WHEY**	**YMP**	**SBM**	**WHEY**	**YMP**		
**Time 4 h**												
Acetic	17.0^d^	17.2^d^	17.2^d^	17.2^d^	18.1^bc^	17.3^d^	17.5^cd^	19.2^a^	17.6^cd^	18.9^ab^	0.178	0.000
Propionic	5.88	5.89	5.94	5.94	6.06	5.92	5.94	6.06	6.03	5.97	0.060	0.380
Butyric	1.90^c^	1.90^bc^	1.92^bc^	1.93^bc^	1.93^bc^	1.91^bc^	1.98^b^	1.93^bc^	1.94^bc^	2.18^a^	0.018	0.000
Valeric	0.16	0.18	0.15	0.16	0.15	0.16	0.16	0.17	0.16	0.17	0.006	0.338
Total BCVFA	0.34^b^	0.35^b^	0.36^b^	0.35^b^	0.36^b^	0.41^a^	0.37^b^	0.43^a^	0.41^a^	0.41^a^	0.007	0.000
Total VFA	25.3^c^	25.5^bc^	25.6^bc^	25.6^bc^	26.6^ab^	25.7^bc^	26.0^bc^	27.8^a^	26.1^bc^	27.6^a^	0.252	0.000
Lactic	5.16^d^	5.23^d^	5.15^d^	5.33^d^	6.55^b^	5.37^d^	6.29^bc^	9.46^a^	5.57^cd^	9.98^a^	0.170	0.000
**Time 10 h**												
Acetic	35.8^c^	36.6^bc^	35.7^c^	36.7^bc^	38.9^b^	37.4^bc^	38.0^bc^	44.8^a^	37.2^bc^	42.9^a^	0.518	0.000
Propionic	21.6^de^	22.2^cde^	21.5^e^	22.3^bcde^	24.3^b^	23.3^bcde^	23.6^bcd^	31.0^a^	24.1^bc^	30.8^a^	0.396	0.000
Butyric	6.35^d^	6.54^d^	6.39^d^	6.54^d^	6.67^cd^	6.80^cd^	7.10^bc^	7.53^b^	7.14^bc^	8.76^a^	0.099	0.000
Valeric	0.61^e^	0.69^cde^	0.63^de^	0.67^de^	0.68^cde^	0.74^bcd^	0.76^bcd^	0.85^b^	0.80^bc^	1.28^a^	0.023	0.000
Total BCVFA	0.35^d^	0.39^d^	0.46^c^	0.36^d^	0.39^d^	0.68^b^	0.37^d^	0.34^d^	1.01^a^	0.35^d^	0.010	0.000
Total VFA	64.8^c^	66.4^bc^	64.7^c^	66.5^bc^	70.9^b^	68.9^bc^	69.9^b^	84.5^a^	70.2^b^	84.1^a^	0.994	0.000
Lactic	0.14^b^	0.15^b^	0.13^b^	0.14^b^	0.14^b^	0.17^b^	0.16^b^	0.18^b^	0.16^b^	0.31^a^	0.019	0.000
**Time 24 h**												
Acetic	56.3^de^	55.8^e^	56.2^e^	56.2^e^	59.7^bc^	58.0^cde^	59.3^bcd^	69.6^a^	61.6^b^	67.5^a^	0.623	0.000
Propionic	26.7^d^	27.1^d^	27.2^d^	26.8^d^	30.3^c^	29.2^c^	30.3^c^	39.9^a^	32.4^b^	40.4^a^	0.281	0.000
Butyric	8.81^d^	8.89^d^	8.86^d^	9.02^d^	9.59^c^	9.66^c^	10.1^c^	12.1^b^	11.5^b^	12.8^a^	0.118	0.000
Valeric	3.13^e^	3.15^e^	3.28^de^	3.22^de^	3.58^cde^	3.86^c^	3.72^cd^	4.84^b^	5.46^a^	4.90^b^	0.105	0.000
Total BCVFA	1.25^g^	1.34^fg^	1.49^ef^	1.31^g^	1.89^d^	2.70^c^	1.61^e^	3.76^b^	6.05^a^	1.86^d^	0.034	0.000
Total VFA	96.2^d^	96.3^d^	97.0^d^	96.6^d^	105^c^	103^c^	105^c^	130^a^	117^b^	127^a^	0.923	0.000
Lactic	0.08	0.06	0.08	0.06	0.08	0.06	0.06	0.11	0.05	0.07	0.037	0.994

1 CTR, control; SBM, soybean meal; WHEY, whey protein; YMP, yeast-derived microbial protein; BCVFA, branched-chain volatile fatty acids.

a−g *Means within rows with different superscripts differ significantly (P < 0.05; Tukey's HSD test)*.

#### Relative Yield of Branched-Chain Fatty Acids

**Figure 2:**
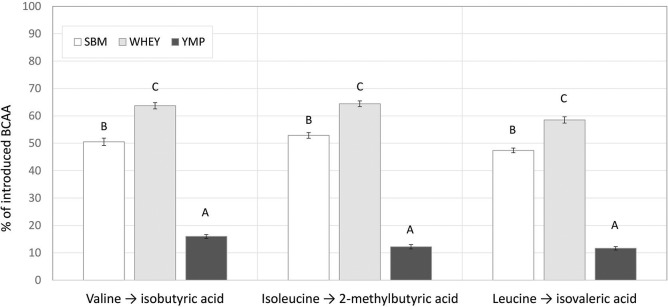
Proportion of introduced branched chain amino acids (BCAA) recovered as the corresponding branched chain volatile fatty acids (BCVFA) after *in vitro* rumen fermentation. Percent conversion of each BCAA to the corresponding BCVFA was estimated by comparing the concentration increase in each BCVFA caused by a protein supplement to the amount of corresponding BCAA introduced in the fermentation vessel with the supplement (see Table 1). The data shown are for fermentation vessels with the highest dose of supplements and 24 h of fermentation. Error bars indicate standard error of the mean. Different superscripts above bars for each acid indicate significant differences between protein supplements (*P* < 0.05; Tukey HSD test).

Although the low, middle, and high doses of the test products were isonitrogenous, the amount of BCAA introduced was different due to the differing amino acid composition of the proteins (Table 1). This difference in composition may partly account for the differences in BCVFA concentration detected in *in vitro* rumen fermentation. Therefore, we also calculated the yield of BCVFA as a percentage of BCAA introduced with the protein supplement. The BCFA produced in the unamended control vessels were subtracted to calculate the percent conversion of BCAA added with the test supplements to corresponding BCFA. Figure 2 shows the percentage of Val, Ile, and Leu recovered as isobutyric, 2-methylbutyric, and isovaleric acid, respectively, after 24 h of fermentation with the high dose of different protein sources. WHEY showed the highest conversion level for all these acids, averaging 62% of amino acids introduced. Thus, according to this model 38% of WHEY protein would be rumen undegradable. For SBM, the apparent recovery of BCVFA was 50%, while for YMP it was 13% (Figure 2). Thus, based on the percent conversion of BCAA to BCVFA, RUP for SBM and YMP would be 50 and 87% of the total protein in each supplement, respectively. At lower doses of the protein supplements, the apparent percentage of RUP decreased to 30 and 70% for WHEY and YMP, respectively, while RUP in SBM remained at the level measured for the high dose (data not shown).

#### Residual Concentration of Ammonia

**Figure 3:**
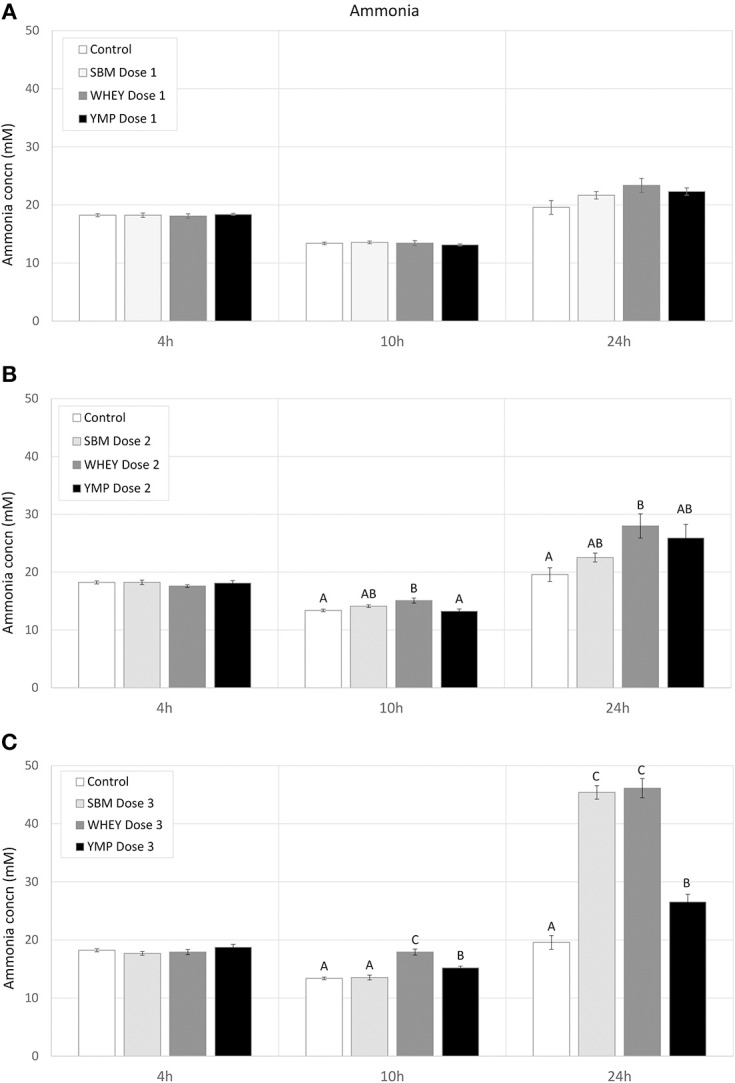
Effect of protein supplements on residual concentration of ammonia in *in vitro* rumen fermentation. **(A–C)** Show the concentration of NH_3_ in treatments with low, medium, and high dose of protein supplement, respectively. Error bars indicate standard error of the mean. Different superscripts above bars at each fermentation time indicate significant differences between protein supplements (*P* < 0.05; Tukey HSD test).

Fermentation of protein (amino acids) susceptible to bacterial degradation produces NH_3_. On the other hand, many multiplying bacteria assimilate NH_3_. Thus, changes in NH_3_ concentration reflect the balance between the rate of amino acid deamination and NH_3_ assimilation. Here, the residual concentration of NH_3_ was analyzed to complement the analysis of BCVFA in assessment of the rate of protein degradation. In all treatments, the residual concentration of NH_3_ dropped slightly between 4 and 10 h of fermentation (Figure 3). However, the NH_3_ concentration increased with the medium and high dose of WHEY compared with the other protein supplements. Between 10 and 24 h, in treatments with protein supplementation the degree of deamination exceeded the degree of bacterial assimilation of NH_3_. The highest residual concentration of NH_3_ was measured in WHEY-supplemented cultures. SMB and YMP differed from each other only at the highest protein dose, when the NH_3_ concentration after 24-h fermentation with YMP was only 60% of that with SBM (Figure 3). This suggests that the rate of deamination was higher for the amino acids in SBM protein than those in YMP protein.

### Effect of Protein Supplementation on Overall Rumen Fermentation Activity

#### Residual Concentration of Short-Chain Fatty Acids

**Figure 4:**
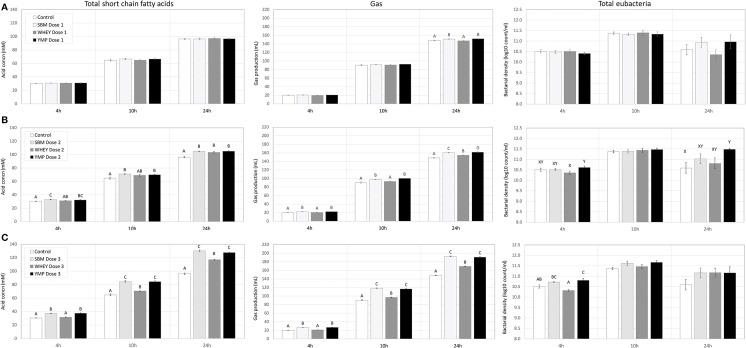
Effect of protein supplements on production of short-chain fatty acids (SCFA), gas, and bacterial cells in *in vitro* rumen fermentation. **(A–C)** Show production of SCFA, cumulative gas, and bacteria in treatments with low, medium, and high dose of protein supplement, respectively. Error bars indicate standard error of the mean. Different superscripts above bars at each fermentation time indicate significant differences between protein supplements at *P* < 0.05 (superscripts A–D) or *P* < 0.10 (superscripts X, Y) (Tukey HSD test).

Rumen bacteria produce lactic acid and VFA as fermentation intermediate and end products, respectively. Therefore, acid concentrations were monitored to assess the activity of bacteria in the fermentation vessels. Lactic acid is a common transient intermediate of carbohydrate fermentation by rumen bacteria. During the first hours of fermentation, the medium and high doses of SBM and YMP increased lactic acid concentration, but this was not the case with WHEY protein (Table 2). At the 10- and 24-h time-points, the concentration of lactic acid was negligible. The concentration of total VFA changed during fermentation from 25 mM at 4 h to 96 mM at 24 h. All protein supplements increased the concentration of total SCFA but SBM and YMP increased it more than WHEY (Table 2; Figure 4). This suggests that the overall stimulatory effect of SBM and YMP on rumen fermentation was higher than the effect of WHEY.

#### Gas Production

In addition to the SCFA data, Figure 4 also shows the effects of the protein supplements on microbial gas production, to enable comparison of the two parameters. Production of gas during fermentation was used as a second parameter for estimation of bacterial activity. Results showed that the response profile of protein supplements in terms of gas production at various doses and time-points was nearly identical to that observed for SCFA (Figure 4). Again, SBM and YMP showed a stronger response for the parameter than WHEY.

#### Bacterial Growth

At the medium and high doses, SBM and YMP increased bacterial density as early as the 4-h time-point (Figure 4). Between the 4- and 10-h time-points, the density of bacteria increased by ~1 order of magnitude, from 3 × 10^10^ to 3 × 10^11^/mL, corresponding to ~3 bacterial doublings. The differences between treatments were no longer significant at the 10-h time-point, but numerical trends were detectable at the high dose of the protein supplements.

### Correlation Between the Fermentation Parameters Analyzed

As fermentation progressed, most of the analyzed metabolites accumulated and after 24 h of fermentation they had a positive correlation with each other. However, during the logarithmic bacterial growth phase prior to the growth plateau, qualitative effects of supplements on fermentation were clearer. Pearson correlation analysis of the parameters show a strong correlation between bacterial density, gas production, and total VFA (Table 3). All these variables are indicators of bacterial growth. The analysis also showed a strong correlation between NH_3_ and BCVFA, both of which are considered indicators of amino acid fermentation. Neither BCVFA nor NH_3_ was correlated with the general bacterial fermentation products or bacterial density (Table 3).

**Table 3:** Correlations between parameters analyzed after 10 h of *in vitro* rumen fermentation^a^.

**Parameter**	**Total VFA**	**Bacterial density**	**Total BCVFA**	**Ammonia**
Cumulative gas	0.936	0.476	−0.227	0.080
	**0.000**	**0.000**	0.080	0.543
	60	59	60	60
Total VFA		0.408	−0.154	0.109
		**0.001**	0.241	0.406
		59	60	60
Bacterial density			−0.068	0.187
			0.611	0.157
			59	59
Total BCVFA				0.736
				**0.000**
				60

a *The three values for each parameter indicate the magnitude and direction of Pearson correlation, statistical significance of the correlation, and number of samples on which the calculation was based, respectively. Significant values (P < 0.05) are shown in bold. BCVFA, branched chain volatile fatty acids*.

## Discussion

Assessment of dietary protein digestibility in the rumen has been of interest for decades, since in ruminants the diet fed is fundamentally modified in the rumen before it is exposed to the digestive system of the host. A detailed understanding of rumen digestion would greatly help professionals in optimizing diets for ruminants at different physiological state. *In vivo* approaches would yield the most realistic data, but surgical operations such as rumen and duodenal cannulation are a prerequisite for analysis of digestibility and microbiota parameters in that case (11). If cannulated animals are available, it is tempting to use *in sacco* methods to monitor microbial degradation of protein. However, the drawbacks reported include physical barriers to microbiota colonization when the test material is in a nylon bag and unsuitability of the method for soluble or small particle size protein supplements which can escape the bag without being degraded (12–14).

To avoid the shortcomings and complexity of *in sacco* methods, various *in vitro* techniques have been developed (15, 16). *In vitro* methods have been widely used for studying overall rumen fermentation activity, but only in few cases for studying protein digestibility (14, 17). Protein fermentation always produces NH_3_, the residual concentration of which varies depending on the rate of bacterial growth and intensity of NH_3_ assimilation (18, 19). Thus, NH_3_ is a marker of protein degradation, but since it is not a terminal end product the residual concentration cannot be used as a single parameter to assess the extent of protein degradation. If the fermenting microbial community is carbon-limited, the lack of carbohydrate-nitrogen synchrony leads to poor microbial protein synthesis and, consequently, accumulation of NH_3_ (20). To sum up, numerous scientific approaches have been developed over the years to examine microbial degradation of protein in the rumen, but no single ideal, affordable, and reliable method for routine use has been found (21–23).

The objective in the present study was to test a new approach for assessing protein degradation by rumen bacteria and, as an example, compare three different protein supplements expected to represent different degrees of resistance to rumen degradation. Even if the method used does not determine the absolute rate of protein degradation in the rumen with optimized diets, it can be used to rank protein sources for their susceptibility to rumen degradation and rumen escape potential. While all amino acids potentially undergo the same reactions, such as deamination and decarboxylation, the metabolic products of most amino acids are the same as those produced in the metabolism of carbohydrates and many other compounds. Therefore, metabolites such as straight-chain VFA, carbon dioxide, and methane cannot be used as a specific indicator of protein fermentation. However, apart from NH_3_ and BCVFA, there are metabolites such as amines and indoles that are specific indicators of bacterial degradation of protein (24, 25). The approach applied here estimates the degree of protein degradation and its rumen bypass characteristics by monitoring residual concentrations of three different BCVFA (isobutyric-, 2-methylbutyric, and isovaleric acid) at three time-points during fermentation. These three BCVFA are bacterial metabolites of the corresponding BCAA (Val, Ile, and Leu). The protein supplements tested had different amino acid composition and BCAA content. The abundance of Val, Ile, and Leu was highest in WHEY, comprising 6.6, 7.1, and 11.6 % of total protein, respectively. Yeast-derived microbial protein had the lowest content of Ile and Leu (3.9 and 6.2%, respectively) and SBM the lowest content of Val (4.6%) (calculated from data in Table 1). Due to the different amino acid composition of the protein sources, a similar concentration of BCVFA produced during fermentation would not indicate an equal percentage of protein degraded for different protein supplements. Therefore, the percentage of protein degraded was calculated for each supplement from the extent of BCAA conversion to the corresponding BCVFA by considering the initial content of Val, Ile, and Leu in the supplement.

There are reports indicating that fermentation by rumen bacteria is stimulated by the addition of BCAA and BCVFA (26–29). Allison et al. (30, 31) showed that some rumen ruminococci indeed require BCVFA for growth. They also showed that isovalerate is used as a carbon skeleton in Leu synthesis and that BCVFA are also incorporated into lipids in the cell wall. However, the concentration of isovalerate required was low, as 0.2 mM was sufficient for maximum growth stimulation and the proportion incorporated was a small fraction of the acid introduced. The findings described above would suggests that some BCAA are likely to be incorporated into microbial protein, and, not all BCVFA produced are necessarily terminal end products. This means that when the system is protein limited these issues would affect the results of protein degradability when calculated from percent conversion of BCAA to BCVFA. With the highest doses of test supplements, we intentionally overdosed the cultures with protein with no concomitant balancing of the system with carbohydrates. Theoretically, protein overdosing reduces the risk of bias caused by incorporation of BCAA and BCVFA into microbial biomass. In the current study such bias was not detected since the percent conversion of BCAA to BCVFA did not decrease with decreasing dose of protein supplements.

We used SBM as a benchmark product in this study, because it is a commonly used protein source and its digestibility has been studied. When the digestibility was calculated based on conversion of three independent BCAA to the corresponding BCVFA, the results were consistent. In 24 h of fermentation, the apparent digestibility of SBM introduced at the highest dose was 50.5, 52.9, and 47.4% when calculated from conversion of Val to isobutyric, Ile to 2-methylbutyric, and Leu to isovaleric acid, respectively. Also, at lower doses of SBM, the apparent digestibility was consistently around 50%, which is in line with the 50–60% protein degradability values reported previously for SBM using different methods (32, 33). Indeed, the consistent results are highly promising when considering potential differences in the composition and processing of SBM products studied, the factors that are assumed to affect degradability (33).

The method applied showed good power in comparing different protein supplements. WHEY protein was the most degradable of those tested, with 62% being degraded in 24 h at the highest product dose and as much as 70% at lower product doses. Protein in YMP was more resistant to degradation by rumen bacteria than that in the other supplements. In 24 h, only 13% of YMP protein added at the highest dose was degraded when measured from recovery of BCVFA. With the lower doses, up to 30% of YMP protein was degraded. The same YMP product has been tested *in vivo* in previous studies to evaluate the effects of replacing some of the SBM in the diet with YMP (34, 35). The aim in those studies was to provide dairy cows with a protein that contains the optimum amino acid profile and low rumen degradability. With 0–3.4 % inclusion of YMP in the diet, a linear negative correlation was observed between the dose of YMP and ruminal parameters, NH_3_, and isovaleric acid. YMP inclusion tended to increase fat- and energy-corrected milk production in high-producing cows (34).

The residual concentration of NH_3_ is not an analyte that could be used in quantitative assessment of the degree of protein degradation. However, in the present study the residual concentration of NH_3_ showed a strong correlation with the concentration of BCVFA (correlation > 0.7; *P* < 0.001). Neither of these parameters was correlated with gas production, total VFA, or bacterial density. This indicates that NH_3_ and BCVFA were true markers of protein degradation, rather than overall activity of rumen fermentation, under the conditions used in this study.

The parameters measuring overall rumen fermentation activity responded differently to the protein supplements tested. Overall activity of rumen bacterial metabolism was measured by gas production, VFA production, and bacterial growth. These three parameters were significantly correlated with each other. Interestingly, WHEY inclusion increased gas and total VFA production only after 24 h of fermentation and/or at the highest dose. Soybean meal and YMP stimulated gas and VFA production at the medium dose and after 10 h of fermentation. The difference between WHEY and other protein sources is most likely due to non-protein components of SBM and YMP, which represent about half of product dry matter. Soybean meal contained readily fermentable protein and also fermentable carbohydrates, which stimulate overall rumen fermentation. The chemical composition of YMP appeared to be very different, with readily fermentable carbohydrates capable of stimulating rumen VFA production and highly resistant protein structures which, *in vivo*, most likely largely escape rumen fermentation.

The results of the study reported here suggest that the approach of following the conversion of BCAA to corresponding BCVFA is an efficient way of monitoring protein degradation by rumen bacteria. In this study, the herd used as a source of rumen fluid had no previous history of feeding the protein sources tested. Thus, the microbiota used as an inoculum was not adapted to degradation of any test protein supplement, eliminating a potential experimental bias. To validate the methodological approach further studies should be conducted using multiple cows as sources of rumen fluid for assessment of animal-to-animal variation. Also, the results of the *in vitro* method described here should be compared with the results of an *in vivo* methodological approach, using exactly the same protein supplements. The relative differences in the resistance of the three protein supplements in the present study were substantial and followed the expected ranking for degradability. Even if the method described in this paper would not give the absolute percent degradability in the rumen, it is accurate when the purpose is to rank protein products, plant varieties, production processes, etc. for protein degradability. A ranking tool would help those formulating diets to meet protein requirements of cows in different conditions or phase of lactation.

## Data Availability

The datasets generated for this study are available on request to the corresponding author.

## Ethics Statement

The animals used as a source of rumen fluid were cannulated and maintained in the research facility of Alimetrics Ltd. in Southern Finland, in accordance with EU Directive 2010/63/EU. The cannulation was approved by the Animal Experiment Board in Finland.

## Author Contributions

All authors contributed to the design of the study and interpretation of results. JA and KV wrote the manuscript. VH and CM provided their valuable comments in the editing phase. KR carried out the chemical analyses.

### Conflict of Interest Statement

JA, KV, and KR are employed by an independent contract research company Alimetrics Ltd., and VH and CM by Alltech corporation whose product was one of the test products used in the present study. Interpretation of results is completely based on scientific criteria and the authors declare no competing interests. The authors declare that this method development project was jointly funded by Alimetrics and Alltech. The involvement of the funders was as follows: Alimetrics and Alltech jointly designed the study and interpreted the results; Alimetrics scientists carried out the actual research work.
